# Long–term Neurodevelopment outcomes to Prenatal Antipsychotic Medication Exposure: Systemic Review

**DOI:** 10.1192/j.eurpsy.2023.1070

**Published:** 2023-07-19

**Authors:** T. González Campos

**Affiliations:** Psychiatry, University Hospital “Dr.José E. González” Universidad Autónoma de Nuevo León, Monterrey, Mexico

## Abstract

**Introduction:**

Peak incidence for many psychiatric disorders occurs in reproductive years. We don’t have FDA-approved drugs or clinical guidelines. The main cause of pharmacological suspension in pregnancy is the lack of knowledge of their effects on the product. Gathering information on this regard becomes crucial so we can avoid relapses or exacerbation

**Objectives:**

Describe neurodevelopmental effects in children ≥ 6 months exposed to antipsychotics prenatally.

**Methods:**

PROSPERO-CRD42020170314. Using MeSH terms in 5 databases, without language and time restriction, obtaining n=3932. After review and exclusion n=24 were obtained for qualitative review. **(Figure 1).**

**Results:**

Of 20 case reports 4 had more ambivalence results **(Table 1).** In the cohort studies, exposure may cause short-term delay at 6 months but no significant difference at 12 months **(Table 2).**

**Table 2:** 

**Study**	**N**	**Antipsychotic**	**Months**	**Conclusion using clinical evaluation**
Imaz et al. Front. Pharmacol.2018; 9:264	1	Risperdal Clozapine	72	**Abnormal** memory, attention/executive, hyperactivity
	1	Clozapine	32	Normal
	2	Clozapine	18	**Abnormal**
				Normal
Burt et al. Am J Psychiatry 2010; 167:892-897	1	Olanzapine	12,18, 22,29	**18 months Abnormal** motor **22 months** normal **29 months** normal
Kirchheiner et al. Pharmacopsych 2000; 33:78-80	1	Olanzapine	7,11	**7 months Abnormal** motor **11 months** Normal
Mendhekar et al. J Neuropsychiatry Clin Neurosci 2007; 19:2	1	Clozapine	6	**5 years Abnormal** language

**Table 3:** 

**Study**	**N**	**Antipsychotic**	**Months**	**Conclusion using clinimetry and clinical evaluation**
Shao et al. Plos ONE, 2015; 10(4),1-9	63	Clozapine Risperidone Olanzapine Quetiapine	6,12	BSID-III **6 months** Adaptive behavior score lower in clozapine group **12 months** No difference
Johnson et al. Arch Gen Psychiatry2012; 69(8), 787-794	22	Haloperidol SGA*	6	INFANIB Lower scores with SGA then Haloperidol
Peng et al. Psychopharmacology 2013; 228(4), 577-584	76	Clozapine Risperidone Sulpiride Olanzapine Quetiapine	6,12	BSID-III **6 months** Lower socio-emotional and adaptive behavior scores **12 months** No difference
Petersen et al. BMJ 2016; 5(6), 1-9	290	FGA+SGA**	6	More neurodevelopmental disorder that those who didn’t take antipsychotics.

**Image:**

**Figure:**
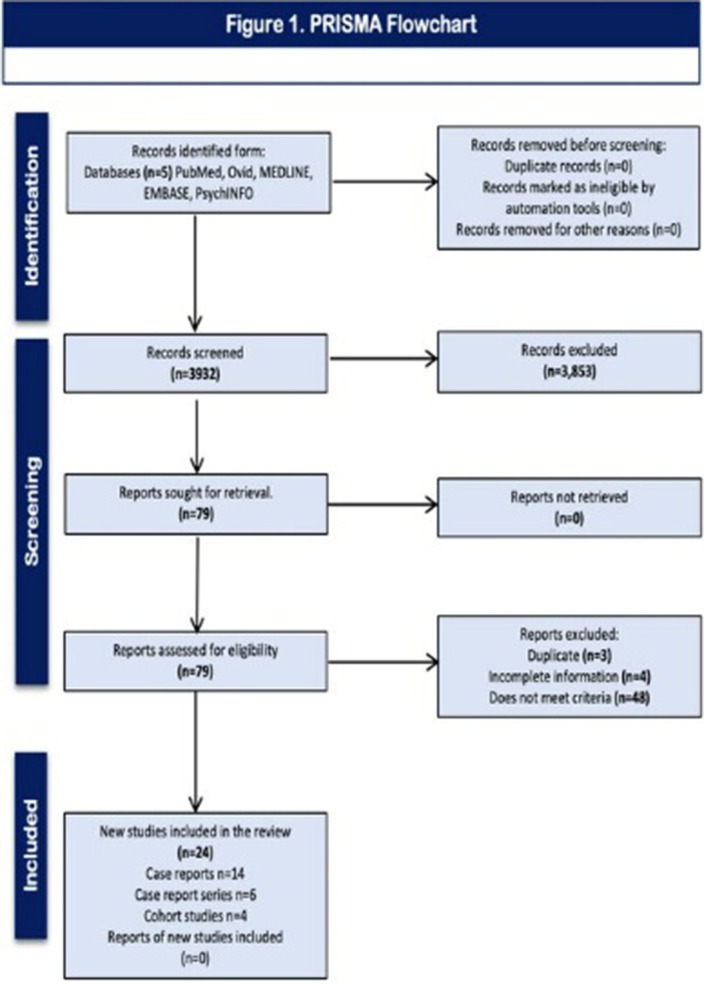

**Conclusions:**

Most are case reports, hence the importance of collecting this information, not ignoring it due to lack of methodological rigor. The intent is not to conclude that prenatal exposure to antipsychotics doesn’t have long-term neurodevelopmental effects, rather documenting the available evidence contributing to an informed clinical decision.

**Disclosure of Interest:**

None Declared

